# Physicochemical, microbial, and functional attributes of processed Cheddar cheese fortified with olive oil–whey protein isolate emulsion

**DOI:** 10.1002/fsn3.3159

**Published:** 2022-12-07

**Authors:** Shafeeqa Irfan, Mian Anjum Murtaza, Ghulam Mueen ud Din, Iram Hafiz, Mian Shamas Murtaza, Sobia Rafique, Kashif Ameer, Muhammad Abrar, Isam A. Mohamed Ahmed

**Affiliations:** ^1^ Institute of Food Science and Nutrition University of Sargodha Sargodha Pakistan; ^2^ Department of Food Science and Technology University of Management and Technology Lahore Pakistan; ^3^ Institute of Chemistry University of Sargodha Sargodha Pakistan; ^4^ Department of Food Science and Technology MNS University of Agriculture Multan Pakistan; ^5^ Post Harvest Research Centre, Ayub Agricultural Research Institute 38000 Faisalabad Pakistan; ^6^ Department of Food Science and Technology, Faculty of Agriculture University of Khartoum Shambat Sudan; ^7^ Department of Food Science and Nutrition, College of Food and Agricultural Sciences King Saud University Riyadh Saudi Arabia

**Keywords:** antioxidant potential, O/W emulsion, olive oil, processed Cheddar cheese, whey protein isolate

## Abstract

Olive (*Olea europaea* L.) has triacylglycerols, phenolics, and other antioxidants in its composition playing significant roles in maintaining health and reducing the onset of diseases. This study aimed to analyze the quality, antioxidant, textural profile, and sensory properties of processed Cheddar cheese fortified with 0%, 5%, 10%, 15%, and 20% (v/w) olive oil–whey protein isolate emulsion during 60 days of storage period. The results showed that processed cheese had significantly higher (*p* < .05) antioxidant activity, and total phenolic and flavonoids contents, whereas nonsignificant increase (*p* > .05) in moisture and acidity while decreasing tendencies in pH, fat, protein, and ash contents. Sensory analysis showed that processed Cheddar cheese with 5% emulsion had higher taste, aroma, texture/appearance, overall acceptability scores, and hardness. Conclusively, results indicated that olive oil–whey protein isolate emulsion could be beneficial for manufacturing and commercializing processed cheeses, analogs, or spreads with improved nutritional value and sensory characteristics.

## Graphical abstract

Present study aimed at preparing processed Cheddar cheese fortified with 0%, 5%, 10%, 15%, and 20% (v/w) olive oil‐whey protein isolate emulsion. Quality, antioxidant, textural profile and sensory properties were analyzed during 60 days of storage. The results showed that processed cheese had significantly higher antioxidant activity, phenolics and flavonoids contents.

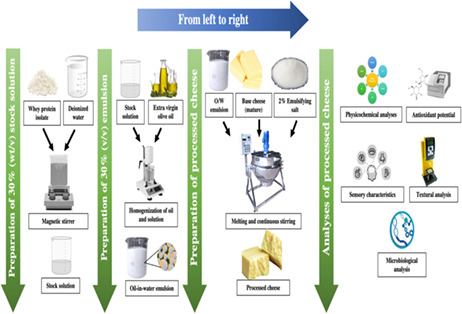

## 1 INTRODUCTION

Processed cheese is a homogenous blend of cheese manufactured from several ingredients, such as the same or different natural cheese varieties, vegetable oils, butter oil, milk solids, emulsifying salts, and other dairy or nondairy ingredients with extended shelf life (Gulzar et al., 2020). Processed cheese has low functionality because of its low nutritional profile, which can be enhanced by incorporating valuable ingredients with high content of bioactive and functional components (Shaukat et al., 2022). Several studies have been conducted to evaluate the influence of different functional materials, such as vegetable powders (El‐Loly et al., 2022), fruits (Abbas et al., 2021), grain flour, and cheese fat substitution through the addition of vegetable oils (Hamdy et al., 2021) on processed cheese.

Olive is grown for olive oil and table olives production in Mediterranean regions (Lanza & Ninfali, 2020). Primarily olive oil is composed of more than 98% triacylglycerols, oleic acid esters, and about 0.5%–1.0% nonglyceridic constituents (Makoś et al., 2017). Moreover, olive oil provides phenolic compounds that are well‐known for their health‐beneficial biological properties. In particular, olive oil's phenolic compounds possess anti‐inflammatory, antioxidant, and antimicrobial activities (Melguizo‐Rodríguez et al., 2021).

Currently, the food industry has a great demand for emulsions (Ranjha et al., 2021). The emulsion is thermodynamically unfavorable (breakdown over time) due to physiochemical mechanisms and can be stabilized using emulsifiers as stabilizers specifically (Weiss et al., 2020). Proteins exhibit amphiphilic character, therefore widely used as emulsifying/stabilizing ingredient in food emulsions. Proteins have the ability to create electrostatic and steric repulsive forces by forming an interfacial layer between oil droplets. During long‐term storage, these forces stabilize the droplets as opposed to coalescence and flocculation (Ding et al., 2021; Marhamati et al., 2021). Whey protein isolates (WPI), an emulsifier, enhance the formation and stability of oil‐in‐water emulsions (Hwang et al., 2017). These also have the capacity to prevent prooxidants to access the droplets, and consequently inhibit lipid oxidation (Nooshkam & Varidi, 2021).

So far, the characteristics and importance of processed cheese fortified with olive oil–whey protein isolate emulsion have not been studied. Therefore, the proposed study was designed to prepare and optimize the emulsion from olive oil with whey protein, improve the functional properties, and assess the physicochemical composition, antioxidant potential, microbiological characteristics, texture, and sensory acceptability of processed cheese fortified with olive oil–whey protein isolate emulsion.

## 2 MATERIALS AND METHODS

### 2.1 Materials

Natural Cheddar cheese (3 months ripened) was procured from Noon Pakistan Ltd. Bhalwal, Sargodha, Pakistan. Extra virgin olive oil was purchased from the Pansari Premium Herbal Store, Lahore, Pakistan. Whey protein isolate (WPI) was purchased online from Myprotein™ (United Kingdom). All other chemicals were obtained from the Dairy Processing Hall, Institute of Food Science and Nutrition, University of Sargodha, Sargodha, Pakistan.

### 2.2 Preparation of olive oil emulsion (OOE)

Extra virgin olive oil and whey protein isolate (WPI) (oil‐in‐water) emulsion was prepared by following the procedure described by Kuhn and Cunha (2012) with some modifications. To prepare WPI stock solution, WPI was dissolved into deionized water at room temperature for 90 min with magnetic stirring and was subjected to overnight storage to achieve complete dissolution at room temperature (25 ± 2°C). The pH of WPI stock solution was adjusted to neutral by using 2.0 M buffer solution (NaOH) and stored at 10°C for a night.

To prepare emulsions, WPI stock solution and olive oil were used in 0.5%:5% (v/v) ratio, respectively. For the preparation of extra virgin olive oil and whey protein isolate emulsion, required amount of olive oil was poured drop‐wise into the required amount of encapsulating WPI solution. The mixture was homogenized at 14000 rpm for 4 min. Sodium azide (0.02% w/v) was mixed into the emulsion. The pH of the emulsions was adjusted to neutral by using 2.0 M buffer solution (NaOH).

### 2.3 Preparation of processed Cheddar cheese

Processed Cheddar cheese was prepared using ripened natural Cheddar cheese, olive oil emulsion (OOE) at 0% (control), 5%, 10%, 15%, and 20% concentrations, and 2% emulsifying salt by following the process described by Kapoor and Metzger (2008), with some modifications. Shredding of the Cheddar cheese was carried out to prepare processed Cheddar cheese. All the ingredients mentioned above were cumulatively poured in a steam‐jacked cooker followed by mixing aided with thermal treatment at a temperature range = 65 to 75°C for a time interval of 15 min. From the cooker, hot‐processed Cheddar cheese was taken out of the molds made with stainless steel having a depth of 10.16 cm. After that, processed cheese samples were cooled to bring their temperature to normal room temperature followed by slicing and packaging. Small rectangular shape blocks were made of processed cheese by slicing and were subjected to packaging under vacuum in polythene bags. Samples were then transferred to the storage facility and processed Cheddar cheese was stored at 2°C for 60 days.

### 2.4 Physicochemical analyses

AOAC (2016) methods were employed for analyzing moisture (AOAC Method No. 948.12), protein (AOAC Method No. 2011.04), ash (AOAC Method No. 942.05), and acidity (AOAC Method No. 942.15) (%lactic acid) contents of processed Cheddar cheese. The fat content of processed Cheddar cheese was evaluated by following the Gerber method with some modifications as given by Marshall (1993). The Gerber method is a volumetric method that employs chemical reagents, such as sulfuric acid to carry out the breakdown of emulsion and fat separation. A special flask was utilized for the measurement of fat content known as butyrometer. Briefly, 10 ml of the pipette was used for transferring sulfuric acid (10 ml) to the butyrometer. The samples were placed in the butyrometer and 1 ml of the amyl alcohol was added using the 1 ml pipette. Then, the butyrometer was placed in the water bath for a time interval of 5 min. After centrifugation, the butyrometers stoppers were oriented downward in the water bath for 3 to 10 min. The difference in the readings was denoted as fat mass in terms of percentage in cheese samples. The pH of processed Cheddar cheese was evaluated for pH by following the method described by Ong et al. (2007) using an Electronic Digital pH Meter. All analyses were carried out thrice (*n* = 3) at the interval of 0, 30, and 60 days during storage.

### 2.5 Preparation of water‐soluble extracts (WSEs)

WSEs of processed Cheddar cheese were prepared by following the method developed by Gupta et al. (2013) with some modifications. Briefly, WSEs were prepared by first mixing 15 g of grated cheese in water (50 ml) followed by mixture placement in the water bath and thermal treatment at a temperature constraint of 40°C for a time interval of 5 min. Then, the mixture was homogenized using Omni‐Mixer homogenizer (Omni International, Waterburg, CT) for a period of 2 min. Furthermore, HCl (2 M) was employed for pH adjustment at 4.6 and then the mixture was added with distilled water until reaching the sample weight (grated cheese sample mixtures) equivalent to 100 g. Then, the placement of the samples in water bath was again carried out at 40°C temperature for 1 h in order to allow the melting fat of cheese samples followed by centrifugation of samples at 4500 rpm (3000 × *g*) at 40°C temperature for 1 h. After centrifugation, Whatman filter paper No. 1 was used for filtration. WSEs of processed Cheddar cheese were collected in a round‐bottom flask and then subjected to freeze‐drying. Powdered freeze‐dried samples were weighed, transferred to plastic tubes, and subjected to storage at −20°C.

### 2.6 Determination of total antioxidant activity

For assessing the ascorbic acid equivalent (AAE) antioxidant capacity, the WSEs were evaluated by following the method described by Prieto et al. (1999) with some modifications at the interval of 0, 30, and 60 days during storage. Briefly, 1 ml of WSEs of processed Cheddar cheese was mixed with 4 ml of phosphomolybdate reagent [28 mM/L NaOH +0.6 M/L H_2_SO_4_ + 4 mM/L (NH_4_)_2_MoO_4_]. The mixture was vortexed for 30 s. Then, it was incubated in the water bath at 95°C for 90 min. The incubated mixture was cooled to room temperature and again vortexed for 30 s. The absorbance of samples was measured at 695 nm through a spectrophotometer (SpectraMax Plus384, Molecular Devices, Sunnyvale, CA). The ascorbic acid‐equivalent antioxidant capacity of processed Cheddar cheese was recalculated using the ascorbic acid standard curve as mg/100 ml AAE. All the replications and experiments were carried out thrice (*n* = 3).

### 2.7 Determination of total phenolic content

For assessing the total phenolic content (TPC) in terms of gallic acid equivalent (GAE), the WSEs of processed Cheddar cheese were evaluated by following the method of Reis et al. (2012) with some modifications at the interval of 0, 30 and 60 days during storage. Briefly, 1 ml of WSEs of processed Cheddar cheese was mixed with 1 ml of 10% (v/v) Folin–Ciocalteu reagent, vortexed for 30 s and left for 10 min at room temperature. Then, 2 ml of 20% (w/v) sodium carbonate (Na_2_CO_3_) was added and again vortexed for 30 s. The mixture was incubated in dark at 30°C for 60 min. After incubation, the absorbance of samples was measured at 760 nm through a spectrophotometer. Gallic acid was dissolved in ethanol and used as a standard. The Gallic acid equivalent (GAE) phenolic content of processed Cheddar cheese was recalculated using the Gallic acid standard curve as mg GAE/100 ml. All the replications and experiments were carried out thrice.

### 2.8 Determination of total flavonoid content

For assessing the total flavonoids content (TFC) in terms of Quercetin equivalent (QE), the WSEs of processed Cheddar cheese were evaluated by following the method described by Zhishen et al. (1999) with some modifications at the interval of 0, 30, and 60 days during storage. Briefly, 1.5 ml of WSEs of processed Cheddar cheese was mixed with 75 μl of 5% (w/v) sodium nitrite (NaNO_3_) and vortexed for 1 min. Then, 150 μl of 10% (wt/v) aluminum chloride (AlCl_3_) solution was added, again vortexed, and left for 5 min. Later, 0.5 ml of 1 M/L sodium hydroxide (NaOH) was added and vortexed again. Afterward, samples were incubated in dark for 45 min. After incubation, the absorbance of samples was measured at 510 nm through a spectrophotometer. Quercetin was dissolved in ethanol and used as a standard. TFC of processed Cheddar cheese was recalculated using Quercetin standard curve as mg QE/100 ml. All the replications and experiments were carried out thrice (*n* = 3).

### 2.9 Microbiological analysis

The total plate count of processed Cheddar cheese was evaluated by following the method of APHA (1984). Bismuth sulfite agar (Hi Media Ltd.) was used for the analysis. Briefly, 10 g of cheese sample was taken in a presterilized pestle and mortar and properly mixed with 90 ml of 0.1% sterile peptone water. Furthermore, 0.1% peptone water was used to prepare 10‐fold serial dilutions. To observe the aseptic conditions, the preparation of samples and serial dilutions were carried out inside the laminar flow cabinet.

### 2.10 Textural profile analysis

Processed Cheddar cheese was evaluated for the texture profile analysis to assess the effect of olive oil–whey protein isolate emulsion and emulsifying salt on the texture of processed cheese by using TA‐XT plus Texture Analyzer and P‐75 compression plate probe by following the method described by O'Mahony et al. (2005). Cheese samples were packed in air‐tight plastic bags and equilibrated for 18 h at 8°C. Samples were cut into cubes of 25 mm height, length, and width through a stainless‐steel wire cutter; before analysis, again equilibrated for 30 min at 8°C. Samples were taken out from the incubator and straightaway compressed in two consecutive cycles at 1 mm/s rate to 30% of the original cheese height.

### 2.11 Sensory evaluation

Sensory quality properties in terms of sensory characteristics of processed Cheddar cheese, such as color, texture, taste, flavor, appearance, and overall acceptability, were evaluated during storage using a 9‐point Hedonic scale. A sensory panel comprising 15 semi‐trained panelists carried out the sensory analysis of processed Cheddar cheese, and panelists included faculty members and post‐graduate students of the Institute of Food Science and Nutrition, University of Sargodha, Sargodha, Pakistan. Moreover, the necessary ethical approval for sensory evaluation from the Institutional Review Board (IRB) was sought under IRB No. SU/IFSN/IRB/004.

### 2.12 Statistical analysis

Data were statistically analyzed using Statistix 8.1 software (analytical software, Tallahassee, Florida, USA). The two‐way Analysis of variance (ANOVA) technique was used to compare the means. A probability of *p* < .05 was used to establish statistical significance. Data were expressed as means ± SD.

## 3 RESULTS AND DISCUSSION

### 3.1 Physicochemical parameters

The moisture, fat, protein, and ash contents of processed Cheddar cheese samples were increased with the corresponding increase in the concentration of OOE. The change in the composition of processed cheese samples was highly significant (*p* < .05) with the addition of OOE. However, nonsignificant (*p* > .05) change was observed in the moisture, fat, protein, and ash contents of processed Cheddar cheese during 60 days of storage because each sample was air‐tightly sealed separately (Table 1). Gab‐Allah (2018) reported that during storage, moisture content of cheese was slightly decreased (*p* > .05) from different treatments that resulted in increased fat and moisture content of the cheese fortified with olive oil and sunflower oil wax. Khaliq et al. (2021) and Shekhar et al. (2015) reported a similar trend in the contents of moisture, fat, and protein of cheese fortified with olive oil during storage. Khaliq et al. (2021) employed extra virgin olive oil (EVOO) for the fortification of cream cottage cheese and concluded that a rise in total volatile fatty acids may be ascribed to protein breakage which leads to the enhanced flavor of cheese product. This enhanced flavor effect was related to moisture content replacement and high discharge of whey protein from the cottage cheese matrix.

**TABLE 1:** Effect of olive oil emulsion on physicochemical parameters of processed cheese

Treatments	Storage period (days)		
	0	30	60
**Moisture (%)**			
**T**_**0**_ (control)	38.25 ± 0.03^L^	38.52 ± 0.04^m^	38.64 ± 0.09 ^n^
**T**_**1**_ (5% OOE)	38.68 ± 0.04^j^	38.81 ± 0.05^k^	38.91 ± 0.07^L^
**T**_**2**_ (10% OOE)	38.96 ± 0.02^g^	39.04 ± 0.01^h^	39.14 ± 0.03^i^
**T**_**3**_ (15% OOE)	39.96 ± 0.03^d^	40.25 ± 0.07 ^e^	40.32 ± 0.05^f^
**T**_**4**_ (20% OOE)	40.47 ± 0.05^a^	40.63 ± 0.02^b^	40.75 ± 0.04^c^
**Fat (%)**			
**T**_**0**_ (control)	30.57 ± 0.02^m^	30.51 ± 0.05 ^n^	30.45 ± 0.03°
**T**_**1**_ (5% OOE)	30.71 ± 0.03^j^	30.65 ± 0.07^k^	30.60 ± 0.02^L^
**T**_**2**_ (10% OOE)	30.98 ± 0.08^g^	30.93 ± 0.02^h^	30.87 ± 0.01^i^
**T**_**3**_ (15% OOE)	31.23 ± 0.04^d^	31.18 ± 0.02 ^e^	31.12 ± 0.02^f^
**T**_**4**_ (20% OOE)	32.56 ± 0.02^a^	32.50 ± 0.03^b^	32.45 ± 0.02^c^
**Protein (%)**			
**T**_**0**_ (control)	26.62 ± 0.06^L^	26.59 ± 0.05^m^	26.56 ± 0.03 ^n^
**T**_**1**_ (5% OOE)	26.84 ± 0.02^j^	26.81 ± 0.06^k^	26.79 ± 0.02^k^
**T**_**2**_ (10% OOE)	27.08 ± 0.03^g^	27.05 ± 0.01^h^	27.02 ± 0.08^i^
**T**_**3**_ (15% OOE)	27.30 ± 0.01^d^	27.28 ± 0.02 ^e^	27.25 ± 0.07^f^
**T**_**4**_ (20% OOE)	27.53 ± 0.05^a^	27.50 ± 0.03^b^	27.48 ± 0.04^c^
**Ash (%)**			
**T**_**0**_ (control)	3.95 ± 0.03^m^	3.92 ± 0.03 ^n^	3.89 ± 0.03°
**T**_**1**_ (5% OOE)	4.60 ± 0.05^j^	4.56 ± 0.03^k^	4.53 ± 0.03^L^
**T**_**2**_ (10% OOE)	4.74 ± 0.02^g^	4.70 ± 0.04^h^	4.66 ± 0.07^i^
**T**_**3**_ (15% OOE)	4.90 ± 0.04^d^	4.87 ± 0.02 ^e^	4.84 ± 0.05^f^
**T**_**4**_ (20% OOE)	4.99 ± 0.03^a^	4.95 ± 0.04^b^	4.93 ± 0.03^c^
**pH**			
**T**_**0**_ (control)	5.84 ± 0.03^c^	5.86 ± 0.06^b^	5.87 ± 0.02^a^
**T**_**1**_ (5% OOE)	5.81 ± 0.02^de^	5.82 ± 0.05^d^	5.84 ± 0.07^c^
**T**_**2**_ (10% OOE)	5.79 ± 0.03^fg^	5.80 ± 0.06^ef^	5.81 ± 0.03^def^
**T**_**3**_ (15% OOE)	5.77 ± 0.02^ij^	5.78 ± 0.04^hi^	5.79 ± 0.05^gh^
**T**_**4**_ (20% OOE)	5.72 ± 0.06^L^	5.75 ± 0.05^k^	5.76 ± 0.05^jk^
**Titratable acidity (% lactic acid)**			
**T**_**0**_ (control)	0.9062 ± 0.0007^L^	0.9045 ± 0.0005^m^	0.9034 ± 0.0003 ^n^
**T**_**1**_ (5% OOE)	0.9215 ± 0.0005^i^	0.9116 ± 0.0002^j^	0.9089 ± 0.0002^k^
**T**_**2**_ (10% OOE)	0.9302 ± 0.0005^f^	0.9285 ± 0.0005^g^	0.9242 ± 0.0003^h^
**T**_**3**_ (15% OOE)	0.9401 ± 0.0005^d^	0.9323 ± 0.0009 ^e^	0.9311 ± 0.0010^f^
**T**_**4**_ (20% OOE)	0.9521 ± 0.0012^a^	0.9499 ± 0.0010^b^	0.9427 ± 0.0015^c^

*Note*: Values are expressed as mean ± SD; means with different letter superscripts are significantly different (*p* < .05).

Abbreviation: OOE = olive oil emulsion.

The pH of the processed Cheddar cheese treatments observed a significant reduction (*p* < .05) with the addition of OOE, which could be due to the carboxylic group of the emulsion. However, the pH insignificantly (*p* > .05) changed during the storage period of 60 days (Table 1). Khaliq et al. (2021) found that the decrease in pH of cream cottage cheese fortified with extra virgin is associated with high acidity. Abbas et al. (2015) reported a decrease in the pH of cheese yogurt fortified with EVOO during cold storage. Shan et al. (2011) reported that the hindrance in pH increase of processed during storage is associated with the higher quantity of phenolic compounds available in the herbal extracts. The acidity of the processed Cheddar cheese increased with increasing the OOE but acidity had a slight decrease (*p* > .05) during storage (Table 1). Shan et al. (2011) reported pH at the initial stage in the range 5.42–5.58 during 9 days of storage, and the authors reported a significantly rising tendency in the pH of control samples.

### 3.2 Antioxidant activity

The results of WSEs of processed cheese are given in Table 2 which ranged from 88.98 mg GAE/100 ml to 107.92 mg GAE/100 ml. However, olive oil provides no less than 30 phenolic compounds (Musumeci et al., 2013). WSEs of processed Cheddar cheese showed a reduction in phenolic content (*p* < .05) during storage. However, phenolic content was higher in WSEs of processed Cheddar cheese as compared to control directly after the production (Table 2). During storage, WSEs of control processed Cheddar cheese did not show a significant change. Peptides that are naturally produced in cheese (Murtaza et al., 2022), due to the activity of starter and rennet, may also act as phenolic compounds. On other hand, some of these peptides react with phenolic compounds present in the cheese to neutralize as well as inhibit their activity (Fox et al., 2004). These peptides may have interacted with the phenolic content of OOE and could describe the reason for the phenolic content decrease in the WSEs of processed Cheddar cheese. The results were according to the findings of Solhi et al. (2020b) in the processed cheese containing asparagus powder. The authors concluded that decreeing tendency in the antioxidant activity of fortified processed cheese samples during storage. This may be attributed to the interactions of phenolic compounds of asparagus and whey proteins with active groups of –SH. Such interactions may lead to a reduction in antioxidant activity. Several enzymes, such as tyrosinase, are usually produced owing to the action of starters, which may cause the conversion of polyphenols into chemical compounds named quinine, which may interact with enzymes and proteins. These secondary reactions may cause alteration of the qualitative and functional properties of proteins even in the sensory characteristics of food products (Solhi et al., 2020a). Fadavi and Beglaryan (2015) reported similar results that UF‐Feta cheese enriched with peppermint extract did not show the expected results of water‐soluble phenolic content due to the rennet concentration.

**TABLE 2:** Effect of olive oil emulsion on the antioxidant potential of processed cheese

Treatments	Storage period (days)		
	0	30	60
**Total phenolic content (mg GAE/100 ml)**			
**T**_**0**_ (control)	88.98 ± 0.03^i^	88.56 ± 0.01^g^	88.34 ± 0.01^j^
**T**_**1**_ (5% OOE)	89.29 ± 0.01^h^	78.89 ± 0.01^m^	64.36 ± 0.06°
**T**_**2**_ (10% OOE)	93.94 ± 0.07^f^	82.92 ± 0.10^L^	76.39 ± 0.10 ^n^
**T**_**3**_ (15% OOE)	101.66 ± 0.04^d^	96.78 ± 0.01 ^e^	85.94 ± 0.03^k^
**T**_**4**_ (20% OOE)	124.42 ± 0.01^a^	116.72 ± 0.01^b^	107.92 ± 0.01^c^
**Total antioxidant content (mg AAE/100 ml)**			
**T**_**0**_ (control)	108.74 ± 0.03^k^	108.69 ± 0.01^k^	108.64 ± 0.02^k^
**T**_**1**_ (5% OOE)	112.51 ± 0.01^j^	115.62 ± 0.01^i^	107.11 ± 0.10^L^
**T**_**2**_ (10% OOE)	117.12 ± 0.01^h^	122.24 ± 0.21^g^	116.78 ± 0.05^h^
**T**_**3**_ (15% OOE)	220.56 ± 0.04 ^e^	227.30 ± 0.03^d^	209.43 ± 0.11^f^
**T**_**4**_ (20% OOE)	303.71 ± 0.01^b^	308.94 ± 0.02^a^	302.11 ± 1.74^c^
**Total flavonoid content (mg QE/100 ml)**			
**T**_**0**_ (control)	43.46 ± 0.02^m^	45.88 ± 0.01^k^	44.89 ± 0.01^L^
**T**_**1**_ (5% OOE)	51.35 ± 0.09^i^	44.89 ± 0.09^L^	33.81 ± 0.06 ^n^
**T**_**2**_ (10% OOE)	62.14 ± 0.05^f^	54.79 ± 0.08^h^	46.78 ± 0.09^j^
**T**_**3**_ (15% OOE)	73.51 ± 0.01^c^	67.98 ± 0.01 ^e^	60.54 ± 0.01^g^
**T**_**4**_ (20% OOE)	83.71 ± 0.01^a^	76.21 ± 0.02^b^	69.83 ± 0.12^c^

*Note*: Values are expressed as mean ± SD; means with different letter superscripts are significantly different (*p* < .05).

Abbreviation: OOE = olive oil emulsion.

Emulsions stabilized with whey protein isolate (WPI) play the role of the antioxidant system, as α‐lactalbumin and β‐lactoglobulin are its major constituents having thiol function, disulfide bonds, and cysteyl residues, which are able to inhibit lipid oxidation by scavenging free radicals (Cayot & Lorient, 1997). Many in vitro and in vivo studies have reported that cheese naturally possesses various biologically active compounds, such as conjugated linoleic acid (CLA), γ‐aminobutyric acid (GABA), vitamins, organic acids, fatty acids, exopolysaccharides, and peptides, exhibiting antiproliferative, antimicrobial, and antioxidant activities and inhibiting angiotensin‐converting enzyme (ACE) (Geurts et al., 2012).

The total antioxidant capacity (TAC) of produced processed Cheddar cheese' WSEs samples are given in Table 2, was in the range of 108.74 mg/100 ml AAE to 168.82 mg AAE/100 ml. Processed Cheddar cheese' WSEs having OOE had significantly higher (*p* < .05) TAC levels compared to control WSEs of processed Cheddar cheese that gradually decreased during 60 days of storage. The decrease in TAC of WSEs of processed Cheddar cheese could be associated with the absorption of phenolic compounds of OOE with proteins' active groups; which might possess the ability to reduce the antioxidant effect of phenolic compounds. Shahidi and Naczk (2003) reported that the polyphenols are turned into quinines due to the activity of enzymes produced by the starters presented in cheese. Quinines are very active and might react with proteins resulting in the change in proteins' nutritional and physicochemical characteristics as well as in the sensory characteristics of food materials. The results are according to the findings of Apostolidis et al. (2007), who reported that herbal extract‐supplemented cheeses had significantly (*p* < .05) higher antioxidant activity in comparison to nonenriched samples. Solhi et al. (2020a) found similar results in their study on processed cheese containing tomato powder. During storage, the antioxidant activity of processed cheese samples exhibited diminished tendency, which could be possibly ascribed to the interaction of protein active groups with phenolic molecules owing to phenolic compounds absorption. In another study on cheese supplemented with extract of dehydrated cranberry fruit, Khalifa and Wahdan (2015) found a decrease in lipolysis, proteolysis, and acid value along with an increase in oxidation stability of the cheese.

The TFC of produced Cheddar processed cheese WSEs samples, given in Table 2, was in the range of 43.46 mg QE/100 ml to 51.74 mg QE/100 ml. WSEs of processed Cheddar cheese showed a reduction in flavonoid content (*p* < .05) during storage. The maximum total flavonoid content (83.71 mg QE/100 ml) was present in T_4_ having 20% OOE at day 0 than control and other treatments. However, T_0_ showed the presence of TFC (43.46 mg QE/100 ml) but content was much less. In dairy products, the antioxidant activity of flavonoids is little known, however, flavonoids' antioxidant activity has been reported (Nadeem et al., 2013). Fruit or plant extracts/ oils may be the source of a higher quantity of flavonoids in any dairy products. Qureshi et al. (2019) reported similar results in a soft cheese (paneer) supplemented with the extracts of date (*Phoenix dactylifera L*.) cultivars and its whey. Authors have also concluded that rich amounts of flavonoids in plant extracts may lead to high concentrations of flavonoids in dairy products or extracts.

### 3.3 Total plate count

Processed Cheddar cheese samples showed a significant (*p* < .05) increase in total plate count during 30 days of storage in control as well as in processed Cheddar cheese containing OOE. Singh et al. (2015) reported a similar decrease in the results of total plate count for the chevon cutlets treated with clove oil. Processed cheese samples having OOE showed a significant (*p* < .05) increase in total plate count throughout 30 days of storage period, but results were significantly (*p* < .05) less compared to the control during storage study. Comparatively, a slow increase in the total plate count in processed Cheddar cheese containing OOE may be credited to the antimicrobial properties of extra virgin olive oil in OOE, as shown in Table 3. Librán et al. (2013) also observed a similar antimicrobial effect of different aromatic plants' aqueous extracts in cheese. Authors have also reported that cheese microbial quality is dependent on unhygienic conditions during manufacturing, postmanufacturing conditions (handling, storage, and packaging), milk thermal treatment, and milk quality. Authors also reported that thermal treatment of milk at 82°C for 5 min time period could lead to the destruction of yeasts and molds as well as coliforms, but postmanufacturing conditions may also lead to microbial contamination (Librán et al., 2013; Qureshi et al., 2019). Mahajan et al. (2015) conducted a study on cheese fortified with pomegranate rind extract and reported similar antimicrobial effect results in the cheese. Comparatively, a slowly rising tendency in the total plate count of fortified cheese samples might be ascribed to the antimicrobial properties of pomegranate rind extract.

**TABLE 3:** Effect of olive oil emulsion on total plate count (log cfu/g) of processed cheese

Treatments	Storage period (days)				
	0	7	14	21	30
**Total plate count (log cfu/g)**					
**T**_**0**_ (control)	3.07 ± 0.08^a^	3.68 ± 0.03^a^	4.32 ± 0.03^b^	4.74 ± 0.04^c^	6.34 ± 0.05^d^
**T**_**1**_ (5% OOE)	2.86 ± 0.02^a^	3.36 ± 0.04^b^	4.03 ± 0.06^c^	4.48 ± 0.06^c^	6.07 ± 0.06^d^
**T**_**2**_ (10% OOE)	2.53 ± 0.05^a^	3.14 ± 0.08^b^	3.73 ± 0.05^c^	4.26 ± 0.05^c^	5.62 ± 0.04^d^
**T**_**3**_ (15% OOE)	2.07 ± 0.03^a^	2.89 ± 0.03^b^	3.58 ± 0.05^c^	3.96 ± 0.07^c^	5.17 ± 0.03^d^
**T**_**4**_ (20% OOE)	1.69 ± 0.05^a^	2.62 ± 0.09^b^	3.27 ± 0.04^b^	3.63 ± 0.03^c^	4.85 ± 0.02^d^

*Note*: Values are expressed as mean ± SD; means with different letter superscripts are significantly different (*p* < .05).

Abbreviation: OOE = olive oil emulsion.

### 3.4 Textural properties of processed cheese

Quigley et al. (2011) reported that texture, a critical characteristic, is the resultant of chemical and physical properties that define the attributes and identity of any food product certainly for the cheese. Processing and compositional parameters greatly affect the textures of cheeses. Hardness has a critical role in the development of cheese texture as Meullenet and Gross (1999) have defined hardness as the required force to bite a sample, positioned in the middle of molar teeth, completely through it. Table 4 shows the hardness of sliced processed Cheddar cheese evaluated with respect to different concentrations of OOE at day 0. The maximum hardness was reported in T_1_ (3800 ± 31.17 g) having 5% of OOE. T_0_ (control), T_1_ (5% OOE), T_2_ (10% OOE), T_3_ (15% OOE), and T_4_ (20% OOE) exhibited springiness values of 0.61%, 0.59%, 0.36%, 0.39%, and 0.28%, respectively. T_0_ (control), T_1_ (5% OOE), T_2_ (10% OOE), T_3_ (15% OOE), and T_4_ (20% OOE) exhibited gumminess values of 1.07, 1.34, 1.65, 1.84, and 1.33 N, respectively (Table 4). These results were similar to findings reported by Sołowiej et al. (2014), who prepared processed cheese using acid and rennet casein at concentrations ranging 11%–13%. Furthermore, the authors investigated the effects of acid and rennet casein substitution on hardness, cohesiveness, and adhesiveness. Processed cheese prepared with the addition of 10% rennet casein and 3% whey protein isolate exhibited a high degree of hardness of 8869.6 g. This increase in hardness might be attributable to the calcium ions' complementary effect on the hydration process as well as para‐casein aggregation, which exerted significant influence on the water‐binding ability of the casein matrix owing to calcium and disulfide bridges formation in conjunction with casein cross‐linkages (Kapoor & Metzger, 2008). Khan et al. (2019) reported similar texture evaluation results. The authors evaluated the effect of *Citrus reticulata* Blanco crude flower extract on processed cheese. The processed cheese added with 2% and 4% crude flower extracts exhibited soft and semi‐hard textures, respectively.

**TABLE 4:** Effect of olive oil emulsion on texture properties of processed cheese

Parameter	0 day				
	T_0_ (control)	T_1_ (5% OOE)	T_2_ (10% OOE)	T_3_ (15% OOE)	T_4_ (20% OOE)
**Hardness (g)**	3857 ± 30.04^d^	3800 ± 31.17^c^	3775 ± 73.90^b^	3745 ± 48.50^a^	3710 ± 49.06^a^
**Springiness (%)**	0.61 ± 0.02^a^	0.59 ± 0.16^d^	0.36 ± 0.06^b^	0.39 ± 0.08^c^	0.28 ± 0.08^a^
**Gumminess (N)**	1.07 ± 0.07^a^	1.34 ± 0.03^c^	1.65 ± 0.23^b^	1.84 ± 0.18^a^	1.33 ± 0.26^d^

*Note*: Values are expressed as mean ± SD; means with different letter superscripts are significantly different (*p* < .05).

Abbreviation: OOE = olive oil emulsion.

### 3.5 Sensorial attributes of processed cheese

Sensory evaluation of different attributes revealed that processed Cheddar cheese treatments having OOE had higher sensory scores of for taste, aroma, texture/appearance, and overall acceptability in comparison to the control (Table 5). The best score for taste (7.65 ± 0.41), aroma (7.13 ± 0.26), texture/appearance (7.50 ± 0.41), and overall acceptability (7.80 ± 0.26) were observed for 5% OOE processed Cheddar cheese during 60 days of storage. In processed Cheddar cheese, the taste is the major determinant for quality and customer acceptance. Control was without OOE, resulting in a lightly salty taste. While, cheese made with 5% and 10% OOE resulted in “slight olive,” “buttery,” and “salty” taste. However, cheeses made with 15% and 20% OOE resulted in “slightly bitter and sour” taste. Similar scores were observed by Caspia et al. (2006). The aromas of the product are very sensitive to processing and storage and influence the flavors of food. Control resulted in typical Cheddar cheese aroma while processed Cheddar cheese having 15% and 20% OOE resulted in high‐intense olive oil aroma. On the other hand, processed Cheddar cheese having 5% and 10% OOE resulted in a less intense olive oil aroma. Khan et al. (2019) reported similar sensory evaluation results. The authors evaluated the effect of *Citrus reticulata* Blanco crude flower extract on the sensory properties of processed cheese. The processed cheese added with 2% and 4% crude flower extract exhibited bitter and umami tastes, respectively. The probable reason for the bitter and umami taste was attributed to the excessive accumulation of small hydrophobic peptides and masking compounds (salt) in cheese curd. After 12 months of the storage period, the Cheddar cheese texture changed because of accelerated ripening. Market value, quality, and consumer acceptance are critically based on the texture of the food product. Results showed that processed Cheddar cheeses having 15% and 20% of OOE had semi‐soft and crumbly texture/ appearance. On the other hand, results showed that 5% and 10% OOE improved the texture/appearance of processed Cheddar cheese as compared to the control. The results were similar to the study conducted by Prinsloo (2007). The quality, taste, aroma, texture, appearance, and likeness or dislikeness of judges decide the products’ overall acceptance. Judges preferred T_1_ and T_2_ having better taste, aroma, and texture/appearance keeping the typical characteristics of Cheddar cheese. These two treatments are even preferred over processed control (T_0_) due to better sensory scoring.

**TABLE 5:** Effect of olive oil emulsion on sensory characteristics (scores) of processed cheese

Treatments	Storage period (days)		
	0	30	60
**Taste**			
**T**_**0**_ (control)	7.69 ± 0.57^a^	6.81 ± 0.56^cd^	5.98 ± 0.40 ^e^
**T**_**1**_ (5% OOE)	7.65 ± 0.41^a^	7.19 ± 0.35^bc^	6.21 ± 0.28 ^e^
**T**_**2**_ (10% OOE)	7.73 ± 0.42^a^	6.80 ± 0.33^d^	6.26 ± 0.32 ^e^
**T**_**3**_ (15% OOE)	7.25 ± 0.35^b^	6.23 ± 0.22 ^e^	5.68 ± 0.21^f^
**T**_**4**_ (20% OOE)	7.05 ± 0.43^b^	5.97 ± 0.23^ef^	5.21 ± 0.23^g^
**Aroma**			
**T**_**0**_ (control)	7.13 ± 0.26^b‐f^	6.94 ± 0.26^d‐h^	6.74 ± 0.26^fgh^
**T**_**1**_ (5% OOE)	7.81 ± 0.62^a^	7.67 ± 0.65^ab^	7.49 ± 0.62^a‐d^
**T**_**2**_ (10% OOE)	7.65 ± 0.41^ab^	7.52 ± 0.43^abc^	7.37 ± 0.44^a‐e^
**T**_**3**_ (15% OOE)	7.15 ± 0.91^b‐f^	6.98 ± 0.95^c‐g^	6.83 ± 0.91 ^e‐h^
**T**_**4**_ (20% OOE)	6.75 ± 0.68^fgh^	6.56 ± 0.70^gh^	6.42 ± 0.68^h^
**Texture/appearance**			
**T**_**0**_ (control)	7.50 ± 0.41^abc^	7.40 ± 0.41^bc^	7.30 ± 0.41^c^
**T**_**1**_ (5% OOE)	7.79 ± 0.24^a^	7.69 ± 0.24^ab^	7.58 ± 0.25^abc^
**T**_**2**_ (10% OOE)	7.70 ± 0.26^ab^	7.60 ± 0.26^abc^	7.50 ± 0.26^abc^
**T**_**3**_ (15% OOE)	6.65 ± 0.48^d^	6.55 ± 0.47^de^	6.45 ± 0.47^def^
**T**_**4**_ (20% OOE)	6.29 ± 0.48^efg^	6.19 ± 0.48^fg^	6.08 ± 0.50^g^
**Overall acceptability**			
**T**_**0**_ (control)	7.80 ± 0.26^a‐d^	7.70 ± 0.27^b‐e^	7.60 ± 0.29^c‐f^
**T**_**1**_ (5% OOE)	8.15 ± 0.34^a^	8.04 ± 0.34^ab^	7.93 ± 0.34^abc^
**T**_**2**_ (10% OOE)	8.00 ± 0.21^ab^	7.91 ± 0.20^abc^	7.81 ± 0.17^a‐d^
**T**_**3**_ (15% OOE)	7.69 ± 0.53^b‐e^	7.55 ± 0.58^def^	7.41 ± 0.55^efg^
**T**_**4**_ (20% OOE)	7.29 ± 0.48^efg^	7.14 ± 0.53^gh^	7.04 ± 0.53^h^

*Note*: Values are expressed as mean ± SD; means with different letter superscripts are significantly different (*p* < .05).

Abbreviation: OOE = olive oil emulsion.

## 4 CONCLUSIONS

It was concluded that processed Cheddar cheese fortified with different concentrations of olive oil–whey protein isolate emulsion is nutritionally excellent as compared to the control. Antioxidant potential was significantly (*p* < .05) higher in processed Cheddar cheeses fortified with olive oil emulsion in comparison with the control. In addition, processed Cheddar cheese with OOE showed better antimicrobial activity compared to the control. Processed Cheddar cheese with 5% emulsion showed excellent sensory perception. Olive oil–whey protein isolate emulsion could be used to manufacture and commercialize processed cheese, analogs, or spreads with improved nutritional value and sensory characteristics. It is recommended that quantification of bioactive compounds (exopolysaccharides, organic acids, peptides, fatty acids, γ‐aminobutyric acid, and vitamins) extracted from processed Cheddar cheese should be done for a better understanding of biological activities.

## CONFLICT OF INTEREST

The authors declare no conflict of interest.

## Data Availability

The data supporting the conclusions of this article are included in the manuscript.
